# The Influence of Pitch Dimensions during Small-Sided Games to Reach Match Physical and Physiological Demands on the Youth Soccer Players

**DOI:** 10.3390/s23031299

**Published:** 2023-01-23

**Authors:** Alfonso Castillo-Rodríguez, Álvaro Durán-Salas, Jesús Vicente Giménez, Wanesa Onetti-Onetti, Luis Suárez-Arrones

**Affiliations:** 1 Department of Physical Education and Sports, University of Granada, 18011 Granada, Spain; 2 Facultad de Educación, Universidad Internacional de la Rioja, 26006 Logroño, Spain; 3 Department of Sports and Computer, University of Pablo de Olavide, 41013 Seville, Spain

**Keywords:** soccer, small-sided games, competition, physical responses, physiological responses, GPS devices

## Abstract

The aims of this study were to (i) analyze the physical and physiological responses of four matches competition and (ii) to investigate the relationships among three different pitch dimensions of small-sided game (SSG) on the youth soccer players. Fifteen male U19 soccer players (age 17.3 ± 0.5 years, height 175.7 ± 5.6 cm, weight 68.5 ± 8.6 kg, playing experience 7.8 ± 1.4 years) were randomly assigned to three play areas: small (50 m^2^), medium (SSG-m, 150 m^2^) and large (SSG-l, 250 m^2^) area per player including goalkeeper. During the 4-week intervention, both groups performed three sets of 8 min with a passive rest period of 5 min between games. Differences in time-motion characteristics of players were measured with the Global Positioning System and assessed using a repeated measures ANOVA to compare the three game conditions and the magnitude-based inference to evaluate the pairwise comparison effects. The results showed that only the variables distance covered between 7.0–12.9 km·h^−1^ was not statistically significantly different among game conditions (*p* < 0.05; *η* = 0.21; small) and physiological response (i.e., hear rate of playing time spent 85–89% HR_max_) also showed differences (*p* < 0.05; *η =* 0.25; small). The responses in SSG-m and SSG-l established them ass the format sizes ideal for replicating the physical responses during match competition. These findings could provide relevant information for coaches for use adequate pitch size (areas of 150 m^2^ and 250 m^2^) to reach the match-play scenarios found in match competition.

## 1. Introduction

Soccer is a team sport with high-intensity intermittent actions, where performance depends on different technical, tactical, biomechanical, psychological, and physiological factors [1,2,3]. Previous research analyzed the training methodology aspects [4] with the purpose of understanding the training process regard to competition [5], where small-sided games (SSGs) are a method widely used usual in soccer training at all levels or ages, from elite teams to children. SSGs simultaneously allow for the improvement of physical and physiological performance alongside technical and tactical aspects utilizing smaller number of players (compared to 11 vs. 11) on a smaller sized pitch [6,7]. SSGs are commonly used by coaches along the micro-cycle (week planning) [8] with different conditional and technical–tactical objectives [9].

Technological advances of the last years have made it possible to monitor a player’s running activity during training sessions and competition using Global Positioning System (GPS) technology. The external (i.e., total distance covered) and internal loads (such as heart rate [HR]) are registered with reliable and valid devices and therefore, could be used in order to improve the training sessions’ quantification and planning [10]. In these sessions, coaches planned training tasks modifying the dimensions of game (pitch size and playing area) [11,12,13], the number of player involved, pitch format, tactical/technical instructions, limits to ball touches or the type of training (e.g., continuous, intermittent, work-to-rest ratio) [9,14,15], the availability of replacement balls, the presence of the goalkeepers, the encouragement of the coach [16], and particularly the rules. All these factors could vary significantly the physiological [17] and physical [18] responses of players.

Currently, during SSGs the pitch dimensions have become a research interest. Previous investigations have sought to compare the acute demands of pitch size [19,20] based on individual area per player (ApP), [21,22,23] as well as the effect of length-to-width ratio. Previous research showed that SSG in a small pitch size (based on individual ApP) induced higher HR and blood lactate concentration [24] compared to large pitch size, because smaller pitches involved a greater number of accelerations, decelerations, and changes of direction [25], in addition to a greater number of technical and tactical actions. However, greater dimensions could perform higher total distances, distance at high speed, among others [25].

To the authors’ knowledge, the physical and physiological responses of different sizes small (SSG-s), medium (SSG-m), and large (SSG-l) SSGs with ApP of 50 m^2^, 150 m^2^, 250 m^2^, respectively, including goalkeeper with the same number of players and their comparison with official games have not been deeply investigated, although the effect of pitch size has been checked and studied in amateur and professional soccer players [26]. Using the competition as a reference in order to prescribe soccer drills and junior players, our hypothesis is to consider the SSG-l are the most appropriate drills to simulate the demands of competition in adolescence soccer players. This information may be useful to coaches to develop the technical and tactical abilities of the players with similar physical and physiological requirements to the competition [27]. Thus, the aim of this study was to assess the effect of different pitch size (SSG-s, SSG-m, and SSG-l) with dimensions proportional to those carried out in competition on the physical and physiological responses in young male soccer players, and their comparison with those obtained during official games.

## 2. Materials and Methods

### 2.1. Participants

Fifteen U19 soccer players voluntarily participated in this study (age 17.3 ± 0.5 years, height 175.7 ± 5.6 cm, weight 68.5 ± 8.6 kg). All the recruited players were members of the same team that competed at the regional league level in the South of Spain, with an average of 8.4 (±2.1) years of experience in soccer. They had an average of 5.9 (±3.5) seasons of experience at a high competitive level. In addition, they trained an average of 8.2 (±1.7) hours per week 5 days a week (not including competition day). All the players (or tutor for players under 18) were carefully informed about the experiment’s procedures and about the potential risk and benefits associated with participation in the study and signed an informed consent document before being included in the study. This study was approved by the Ethics Committee of the University of Granada (Number 471/CEIH/2018) and followed the guidelines set forth in the Declaration of Helsinki (2013). The inclusion criteria were to have been competing for the last four years and available to attend regular training sessions. These criteria were designed to ensure that the participants had sufficient experience and ensure its homogeneity.

### 2.2. Experimental Procedures

An observational design was used to examine the internal and external loads of young soccer players during SSGs and competitive matches using GPS technology and HR response. The study was carried out during a period of 5 weeks between December and January of the competition season 2017–2018. In total, four training sessions (one per week) and four official matches were registered. Before these evaluation weeks, two sessions (in the first week) were dedicated to familiarizing the players with the GPS devices and the different pitch sizes’ drills. In this study, authors considered that evaluated drills are equivalent terms SSGs. These exercises had characteristics similar to those found during the evaluation process (the training drills were similar to those carried out in the 4 weeks of evaluation (they contained tasks from SSG-s, SSG-m and SSG-l)). The evaluation of training sessions and competition matches were carried out at the same time, preceded by a 15-min standard warm-up. All the SSGs performed during the same session were played with the same lineup of players on each team, and players of different positions and roles integrated the teams. During each training session three drills were played corresponding to the three SSGs situations, i.e., SSG-s, SSG-m and SSG-l. The SSGs lasted 8 min with a 5 min passive break between drills, similar to the protocol established by Casamichana and Castellano [25]. The order of play of the different SSGs varied from one session to the next to assure that fatigue was not an influence. Drinking water during the breaks between SSGs was permitted. During evaluation, the GPS devices were worn by each of the four players. The same data was collected for all playing positions: external defenders (ED), internal defenders (ID), external midfielders (EM), internal midfielders (IM), and forwards (FO) [28].

In order to avoid any potential imbalance among the participating players, certain individual and collective aspects were taken into account in the creation of this study: technical-tactical level, participation in match competitions, and playing position. Following the procedure used in a previous study [25], the technical-tactical level of the player was established according to the subjective evaluation of the coach, who rated the player from 1 (lowest level) to 5 (highest level). The number of minutes played in match competitions (before their inclusion in the study) was also used to categorize the players. The usual playing position of the participants (ED, ID, EM, IM, and FO) [28] was registered. Finally, two comparable teams in terms of the subjectivity of the coach, number of minutes played and playing position were created [29].

### 2.3. Individual Playing Area (ApP)

All SSGs were played outdoors in a soccer field with artificial grass. The technical-tactical nature of the SSGs was always similar to that of a competitive match. They were matches in small spaces, but with the same rules of play. During each SSG two opposing teams consisting of 5 versus 5 players and goalkeepers faced each other. The pitch size was variable, but the relative dimensions (length/width) were maintained. These calculations were carried out through equations of the second degree. The SSG-l had the same ApP that during home matches, however, the main difference is that in official matches (11 × 11) they played at 102 × 54 m and in SSG-l (5 × 5) at 75 × 40 m. For the other formats used (SSG-MSSG-m and SSG-s), the ApP was reduced by ~100 m^2^ and ~200 m^2^, respectively [25]. The goalkeepers were taken into account in the calculation of ApP. The SSGs characteristics are in Table 1.

### 2.4. Exercise Intensity

Exercise intensity was quantified by monitoring HR using the Polar S610i (Polar Electro OY, Finland) devices during SSGs and games. Four zones of exercise intensity were established according to the individual HR_max_ (<75% HR_max_, 75–84% HR_max_, 85–89% HR_max_, >90% HR_max_) [30]. The maximal HR of the players was obtained throughout an incremental field test (the highest 5-s average recorded during the test) [31]. Furthermore, the percentage of time that players spent in each zone of intensity during the SSGs was recorded. The variables analyzed were minimum HR (HR_min_), mean HR (HR_mean_), and HR_max_ for each SSG.

### 2.5. Running Demands Analysis

Speed and distance covered was measured using GPS technology (SPI-PRO, GPSports, Canberra, Australia) with the software Team AMS 1.2. These GPS devices operate at 5 Hz. Their reliability was previously tested [32] with results ranging from 2% to 13% during sprinting with an underestimation of 4% for these distances. The GPS registered the total distance (TD) and distances covered at different speeds during the SSGs and official games. Players’ activities were codified in 5 categories and speed thresholds [29]: walking (0.1–6.9 km·h^−1^), low-intensity running (7.0–12.9 km·h^−1^), medium-intensity running (13.0–17.9 km·h^−1^), high-intensity running (18.0–20.9 km·h^−1^), and sprinting (>21.0 km·h^−1^). The average number of satellites registered during the SSGs was 8 ± 1.

### 2.6. Statistical Analysis

Data are shown as means and standard deviations with a 95% confidence interval. The Levene test was performed for assessment of uniformity of variance, and a one-way ANOVA was performed to determine the differences in dependent variables (physical and physiological responses) and the independent variable was the pitch size (with three different ApP) corresponding to the respective SSGs (SSG-s, SSG-m and SSG-l). The eta-squared values were calculated to estimate effect sizes of ANOVA. Subsequently, if data met the requirements of homoscedasticity, a Bonferroni correction was performed to make comparisons between pairs; if not, a Games-Howell test was performed. The effect sizes were calculated on all comparisons using the following criteria [33]: for ANOVA tests, those values (*η^2^*) were 0.10 for small effects, 0.25 for moderate effects, and 0.40 for large effects. Subsequently, a linear regression analysis (stepwise method) was performed between the physiological responses and the distance covered per minute. SPSS for Windows v.23.0 was the statistical software used. The level of significance was set at *p* < 0.05.

## 3. Results

Table 2 shows the physiological responses of the players during the different pitch size of SSG (SSG-s, SSG-m, and SSG-l) and the official game (first [H1] and second halves [H2]). There were no significant differences between the SSG-s and the official game, except for the playing time spent between 75–84% HR_max_, where differences between SSG-l vs. H2 were detected (*p* < 0.05). Playing time spent between 85–89% HR_max_ was the best physiological variable that explains the distance per minute covered by the players (*R*^2^: 19.2%; SEE: 17.311; *p* < 0.0001).

**Figure 1:**
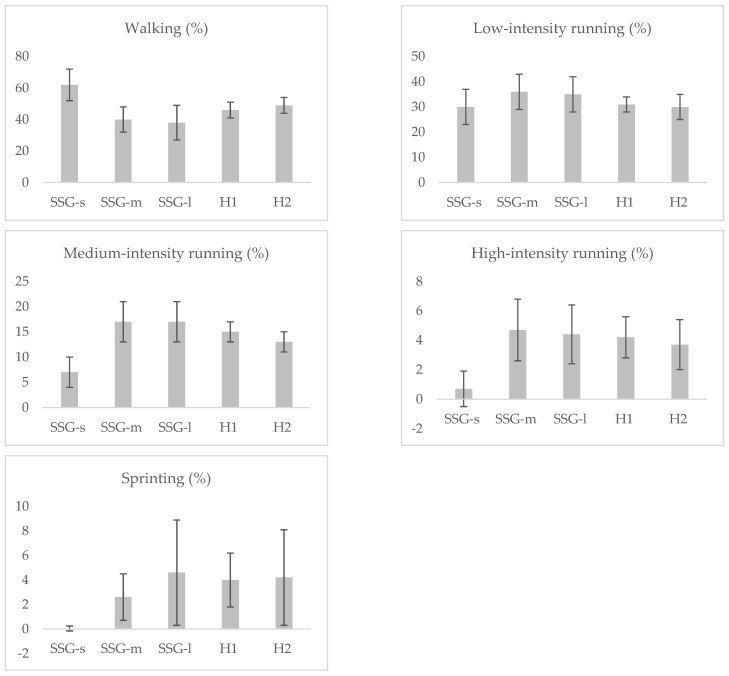
Distances covered to 5 ranges of speed in different SSG and competition match. Walking: distance covered between 0.1–6.9 km·h^−1^; Low-intensity running: distance covered between 7.0–12.9 km·h^−1^; Medium-intensity running: distance covered between 13–17.9 km·h^−1^; High-intensity running: distance covered between 18–20.9 km·h^−1^; Sprinting: distance covered >21 km·h^−1^. SSG-l: small-sided games large; SSG-m (small-sided games medium), SSG-s (small-sided games small). H1: First half; H2: Second half.

Table 3 and Figure 1 show the movement patterns of the players during the different SSG formats (SSG-s, SSG-m, and SSG-l) and the official games (H1 and H2). Absolute total distance and distance covered at different speeds during each half was significantly higher vs. SSGs. Relative total distance covered during the SSG-l was significantly higher vs. SSGs and the official games (*p* < 0.01). Relative total distance covered, and maximal speed reached during the SSG-s was significantly lower vs. SSG-m, SSG-l and the official games (*p* < 0.01). Maximal speed reached during the first half of the game was significantly higher vs. SSG-s and SSG-m (*p* < 0.01, respectively), but with no differences vs. SSG-l. Percentage of playing time running at high intensity and sprinting during each half was significantly higher only vs. SSG-s (*p* < 0.01), with no differences vs. SSG-m and SSG-l.

## 4. Discussion

The aims of this study were to assess the effect of different pitch size (SSG-s, SSG-m, and SSG-l) on the physical and physiological responses in young male soccer players, and to compare these metrics with those obtained during official games. The initial hypothesis was to consider that the SSG-l with similar characteristics and rules as the competition could present similar external and internal load in comparison with the official matches. The results of the present study showed different movement patterns within the different pitch size formats. The results obtained in these SSGs can be transferred and discussed with respect to the demands in the competition study because they are relative data, depending on the playing time. However, information on absolute values has been shown too so that readers can know real values of the demands both in SSGs and in competition. Similar physiological responses were also shown in a previous study that compared the HR responses during SSGs and competition [11]. Our results showed that the running demands during the SSG-s were lower in comparison with SSG-m, SSG-l and official matches, while the movement patterns relative to playing time between SSG-m and SSG-l were similar to the competition, or even most demanding in some parameters evaluated, in the case of SSG-l (i.e., relative total distance). This fact has also occurred in a previous study that compared SSG with 60 and 80 m^2^ of ApP, clearly showing that the second SSG presented greater demands from the players and closer to competition [6]. In this line, previous studies showed that the physical and physiological responses, obtained during SSG-m and SSG-l formats, were consistent with those produced during competition matches [12,34]. In contrast, a previous study found a similar response in all the contexts [13]. Our findings indicated that SSG-l and SSG-m were specific alternatives to running generic exercises in order to train with real demands based on competition. In addition, a previous study demonstrated that a seven-week warm-up training period, using SSGs, improved soccer players’ performance [30].

Previous studies have shown that SSG-m and SSG-l were drills with more similar running demands in comparison with competition [12,34,35]. The most representative variable of the general intensity of the activity was the distance covered per minute (relative distance covered) [25]. Our study showed relative distances covered from 82 to 119 m·min^−1^ for SSG-s and SSG-l, respectively. These results were comparable to those shown by Casamicha et al. [25] with relative distances covered ranged from 87 to 125 m·min^−1^ for SSG-s and SSG-l. In our case, the SSG-m showed similar relative distance covered per minute in comparison to the official matches (first half), with higher relative running demands for SSG-l in comparison with the competition (119 and 103 m·min^−1^, respectively).

The physiological responses shown during the different SSGs in the present study was similar to previous studies [10,27,34], and without statistical differences in comparison with the official matches. In this regard, the SSGs used in this study were useful drills to improve specific endurance in young male soccer players with HR_max_ between 88% for SSG-s to 91% for SSG-l. Previous studies showed similar results with HR_max_ from 86–89% [25,34], 89–91% [11,36], and 91% [37]. Training intensities at or slightly higher than the anaerobic threshold (85–90% HR_max_) appear to be effective for improving the aerobic performance of soccer players [36]. In addition, other studies have demonstrated the ability of SSGs to improve physical and physiological condition after a period of detraining (rest period after the competition season) [38]. Coaches must carefully design their SSGs because these results showed that the internal and external loads experienced by players were affected by the different formats of SSGs. Based on this, we should decide that if we want to simulate the demands of the competition, the physical and physiological response during SSG-s is not the most suitable if you want to train as you compete, being SSG-m and SSG-l more suitable in this case. In this line, a previous study has already described that high-level players do not train as they compete, with large differences (and high effect sizes) in the quantification of training and competition loads [39].

One of the limitations of this study was the sample size, due basically to the relative inaccessibility to high level U19 soccer players. In addition, the availability of four portable devices made it difficult to obtain a greater amount of data, while the number of evaluation days and analyzed matches increased. Therefore, the data collected is rigorous, although not generalizable due to the limited sample size. Despite this limitation, the authors of this study were able to collect and process a series of data that can help coaches to plan more efficient training sessions focused on optimal preparation for competition matches. On the other hand, the subjective perception of the effort could have been assessed in order to compare or relate it to the objective variables of the study evaluated through GPS devices. Furthermore, in light of the findings of this study, there is a need to encourage future researchers to carry out similar studies using a greater number of training sessions in order to evaluate more precisely the coefficient of variation of the physical and physiological responses in relation to competition. As strengths, the study has considered that in SSG-s, players performed more high-intensity activities (i.e., accelerations and decelerations) and could be useful for increasing neuromuscular adaptations during SSG training [5]. Furthermore, it is established for formative soccer, as lines in the teaching methodology that problem solving performed in SSGs with medium and large pitch sizes were similar to those reproduced in competition and could open a wide number of tasks to reproduce, without giving the soccer player a longer decision-making time.

## 5. Conclusions

The main finding of this study showed that for soccer training drills, the ApP should be taken into consideration to get physical and physiological responses similar to those produced during official games. SSGs should incorporate specific aspects about ApP to replicate the demands of competitive matches. If the ApP is 50 m^2^ (SSG-s), only a low level of physical conditioning is achieved. The drills with ApPs most suitable for inducing the physical and physiological responses demanded during competitive matches were 150 m^2^ and 250 m^2^ (SSG-m and SSG-l, respectively). With these drills, soccer players get to train how they are going to compete and reach the match-play scenarios found during official games.

As practical applications, it is recommended that the coaches and physical trainers in soccer design SSG with ApP of at least 150 m^2^ so that those players present greater physical demands in order to optimize these responses during the competition. On the other hand and according to the results of this study, the physiological demands of the players are covered in the three SSGs studied (50 m^2^, 150 m^2^, and 250 m^2^) with respect to the demands of official matches required. Although the physical and physiological demands during SSG-m and SSG-l are closer to competition, all the SSG could produce improvements in fitness and at a technical and tactical level. However, to be adapted to the game, it is necessary to perform drills that, in addition to technical and tactical improvement, provide those demands that are going to be presented in the competition. For these reasons, the pitch size is very useful and appropriate consideration for coaches and technical staff when increasing or decreasing the external load in the soccer players.

## Figures and Tables

**Table 1:** Established characteristics of the three small-sided game formats (SSG-l = large, SSG-m = medium, SSG-s = small) and official matches.

		Small-Sided Game Format		
Variables	Official Matches	SSG-l	SSG-m	SSG-s
Duration	2 × 40 min	8 min	8 min	8 min
Pitch size	102 × 54 m	75.3 × 39.9 m	58.4 × 30.9 m	33.8 × 17.9 m
Playing area	5508 m^2^	3004.5 m^2^	1804.6 m^2^	605 m^2^
Grid ratio	1.9:1	1.9:1	1.9:1	1.9:1
Ratio per player	250.4 m^2^	250.4 m^2^	150.4 m^2^	50.4 m^2^
Goalkeepers	yes	yes	yes	yes
Rules	Rules = soccer 11	Rules = soccer 11		
Coach giving orders	yes	yes	yes	yes
Availability of balls		yes	yes	yes

**Table 2:** One-way ANOVA of the physiological responses on soccer players in different SSG and competition match ^a^. The final column shows the regression coefficient between distance per minute and the physiological metrics.

	SSG-s (n = 15)	SSG-m (n = 15)	SSG-l (n = 15)	H1 (n = 15)	H2 (n = 15)	*F* (4,65)	*p*	*η^2^*	Effect Size Quality	*R*^2^ with TD/Min
HR_min_ (%HR_max_)	53 ± 6	54 ± 7	55 ± 7	48 ± 14	48 ± 16	1.497	0.215	0.15	Small	-
HR_mean_ (%HR_max_)	73 ± 7	78 ± 7	77 ± 8	75 ± 3	73 ± 5	1.627	0.179	0.16	Small	0.160 **
HR_max_ (%HR_max_)	88 ± 8	90 ± 8	91 ± 8	96 ± 8	93 ± 9	1.782	0.144	0.16	Small	0.076 *
HR1	51 ± 33	31 ± 33	33 ± 31	43 ± 14	55 ± 18	1.786	0.144	0.16	Small	0.146 **
HR2	35 ± 24	33 ± 22	28 ± 18	40 ± 10	34 ± 13	0.566	0.689	0.09	Small	-
HR3	10 ± 14	22 ± 16	23 ± 14	11 ± 7	7 ± 8 ^a^	4.294	0.004	0.25	Moderate	0.192 **
HR4	4 ± 2	14 ± 7	14 ± 7	6 ± 3	4 ± 2	2.118	0.090	0.18	Small	0.167 **

^a^ Data are showed as mean ± standard deviation (95% Confidence Interval). HR_min_: minimum heart rate; HR_mean_: mean heart rate; HR_max_: maximum heart rate. HR1: % of playing time spent <75%HR_max_; HR2: % of playing time spent between 75–84%HR_max_; HR3: % of playing time spent 85–89%HR_max_; HR4: % of playing time spent >90%HR_max_. SSG-l: Small-sided games large; SSG-m: Small-sided games medium; SSG-s: Small-sided games small; H1: First half; H2: Second half. a: Substantial difference vs. H1. Linear Regression coefficients (*R*^2^): * *p* < 0.05; ** *p* < 0.01.

**Table 3:** One-way ANOVA of the physical responses on soccer players in different SSG and competition match.

	SSG-s (n = 15)	SSG-m (n = 15)	SSG-l (n = 15)	H1 (n = 15)	H2 (n = 15)	*F* (4,65)	*p*	*η^2^*	Effect Size Quality
TD/min (m/min)	82 ± 12 ^all^	113 ± 13 ^a,e^	119 ± 17 ^a,d,e^	103 ± 11 ^a,c^	98 ± 8 ^a,b,c^	20.237	0.000	0.49	Large
V_max_ (km/h)	18 ± 2 ^all^	24 ± 3 ^a,d^	26 ± 4 ^a^	29 ± 4 ^a,b^	28 ± 5 ^a^	21.467	0.000	0.50	Large
TD (m)	653 ± 95 ^d,e^	906 ± 101 ^d,e^	953 ± 138 ^d,e^	4255 ± 798 ^all^	2839 ± 744 ^all^	159.746	0.000	0.84	Large
Walking (m)	394 ± 26 ^d,e^	354 ± 38 ^d,e^	352 ± 53 ^d,e^	1935 ± 339 ^all^	1398 ± 461 ^all^	131.523	0.000	0.82	Large
LIR (m)	204 ± 75 ^d,e^	328 ± 87 ^d,e^	343 ± 101 ^d,e^	1339 ± 318 ^all^	829 ± 158 ^all^	111.892	0.000	0.80	Large
MIR (m)	50 ± 28 ^all^	157 ± 45 ^a,d,e^	169 ± 55 ^a,d,e^	633 ± 186 ^all^	383 ± 115 ^all^	77.602	0.000	0.76	Large
HIR (m)	5 ± 9 ^d,e^	46 ± 26 ^d,e^	43 ± 23 ^d,e^	180 ± 86 ^all^	110 ± 68 ^all^	27.851	0.000	0.56	Large
Sprinting (m)	0.4 ± 1.5 ^d,e^	24 ± 18 ^d,e^	47 ± 46 ^d,e^	168 ± 105 ^a,b,c^	121 ± 104 ^a,b,c^	15.892	0.000	0.44	Large
Walking (%)	62 ± 10 ^all^	40 ± 8 ^a^	38 ± 11 ^a,e^	46 ± 5 ^a^	49 ± 5 ^a,c^	18.070	0.000	0.47	Large
LIR (%)	30 ± 7	36 ± 7	35 ± 7	31 ± 3	30 ± 5	2.924	0.028	0.21	Small
MIR (%)	7 ± 3 ^all^	17 ± 4 ^a^	17 ± 4 ^a,e^	15 ± 2 ^a^	13 ± 2 ^a,c^	23.474	0.000	0.59	Large
HIR (%)	0.7 ± 1.2 ^all^	4.7 ± 2.1 ^a^	4.4 ± 2 ^a^	4.2 ± 1.4 ^a^	3.7 ± 1.7 ^a^	14.306	0.000	0.42	Large
Sprinting (%)	0.4 ± 0.2 ^c,d,e^	2.6 ± 1.9	4.6 ± 4.3 ^a^	4.0 ± 2.2 ^a^	4.2 ± 3.9 ^a^	6.285	0.000	0.30	Moderate

Data are showed as mean ± standard deviation (95% Confidence Interval). TD: Total distance, V_max_: maximal speed. Walking: distance covered between 0.1–6.9 km·h^−1^; LIR: low-intensity running: distance covered between 7.0–12.9 km·h^−1^; MIR: medium-intensity running: distance covered between 13–17.9 km·h^−1^; HIR: high-intensity running distance covered between 18–20.9 km·h^−1^; Sprinting: distance covered >21 km·h^−1^. SSG-LSSG-l: small-sided games large; SSG-m (small-sided games medium), SSG-s (small-sided games small). H1: First half; H2: Second half. a: Significant difference vs. SSG-s; b: Significant difference vs. SSG-m; c: Significant difference vs. SSG-l; d: Significant difference vs. H1; e: Significant difference vs. H2; all: Significant difference vs. all situations.

## Data Availability

The data presented in this study are available on request from the corresponding author to any qualified researcher, if they have obtained Ethics Approval for secondary use of existing data through a Consent Waiver.
